# Effects of Exercise on Depression and Anxiety in Breast Cancer Survivors: A Systematic Review and Meta‐Analysis of Randomized Controlled Trials

**DOI:** 10.1002/cam4.70671

**Published:** 2025-03-07

**Authors:** Yifan Zhang, Gen Li, Shiyan Zhang, Yilun Zhou, Yuanyuan Lv, Lin Feng, Laikang Yu

**Affiliations:** ^1^ Beijing Key Laboratory of Sports Performance and Skill Assessment Beijing Sport University Beijing China; ^2^ Department of Strength and Conditioning Assessment and Monitoring Beijing Sport University Beijing China; ^3^ School of Physical Education & Sports Science South China Normal University Guangzhou China; ^4^ School of Sport Sciences, Beijing Sport University Beijing China; ^5^ China Institute of Sport and Health Science, Beijing Sport University Beijing China; ^6^ Beijing Sports Nutrition Engineering Research Center Beijing China

**Keywords:** anxiety, breast cancer, depression, exercise

## Abstract

**Background:**

Numerous studies have investigated the effects of exercise on depression and anxiety in breast cancer survivors, yet the results remain inconsistent. The aim of this study was to investigate the effects of exercise on depression and anxiety in breast cancer survivors and to ascertain the optimal exercise regimen for this patient population.

**Methods:**

A comprehensive search was conducted across Embase, PubMed, the Cochrane Library, Web of Science, and Scopus until 28 October 2023. A meta‐analysis was conducted to quantify the standardized mean difference (SMD) and 95% confidence interval (CI).

**Results:**

A total of 25 studies met the inclusion criteria. Exercise significantly alleviated depression (SMD, −0.63, *p* < 0.0001) and anxiety (SMD, −0.49, *p* = 0.0002) in breast cancer survivors. Specifically, aerobic exercise (SMD, −0.44, *p* = 0.03) and multicomponent training (SMD, −0.86, *p* = 0.002) were found to be particularly effective in alleviating depression. However, only multicomponent training significantly alleviated anxiety (SMD, −0.66, *p* = 0.003) in breast cancer survivors. Additionally, multicomponent training conducted for ≥ 3 times per week (depression, SMD, −1.22, *p* = 0.006; anxiety, SMD, −0.93, *p* = 0.004) and ≤ 60 min per session (depression, SMD, −1.19, *p* = 0.002; anxiety, SMD, −0.85, *p* = 0.005) were deemed most effective in alleviating depression and anxiety in breast cancer survivors.

**Conclusions:**

Exercise significantly alleviated depression and anxiety in breast cancer survivors, with multicomponent training being the most effective intervention type. This meta‐analysis provides clinicians with evidence to recommend that breast cancer survivors engage in multicomponent training more than three times per week, with each session lasting no more than 60 min, to alleviate depression and anxiety.

## 1 Introduction

Breast cancer is one of the most prevalent types of cancer among women worldwide. It is estimated that by 2040, there will be over 3 million new cases of breast cancer and 1 million deaths, implying that one in eight women will be diagnosed with the disease in their lifetime [1]. Breast cancer arises from abnormal breast cells growing uncontrollably to form a tumor. It is the primary cause of cancer‐related death among women worldwide [2]. Typical symptoms include breast lumps, nipple discharge, and enlarged lymph nodes in the armpit.

Breast cancer survivors refer to those who have undergone initial treatment, including surgery, radiotherapy, and chemotherapy, and are currently undergoing hormone therapy or are in the follow‐up phase. Throughout diagnosis and treatment, these individuals frequently experience trauma to their self‐image and sexual relationships, leading to psychological reactions like denial, anger, or anxiety, thereby augmenting the risk of mental health issues [3]. They routinely confront physical symptoms, psychological reactions, and survival challenges [4, 5]. Emotional distress is regarded as the sixth vital sign in cancer care, as it can diminish patients' willingness to adhere to treatment plans and increase the risk of mortality [6]. In addition, cancer survivors who suffer from emotional distress often exhibit more severe depression, anxiety, pain, fatigue, and functional impairment and are at an increased likelihood of suicidal thoughts [7].

Recent studies have shown that the prevalence of depression and anxiety in breast cancer survivors is as high as 40%–54.5% and 40%–46.8%, respectively [8, 9]. Studies have identified predisposing factors for these mood disorders, which include a history of depression or anxiety, younger age at diagnosis, inadequate social support, severe somatic symptoms, aggressive cancer treatment, the use of certain medications, fear of death and disease recurrence, as well as changes in body image, femininity, sexual characteristics, and attractiveness [8, 10, 11]. Additionally, studies have shown that patients' symptoms of depression and anxiety may deteriorate during adjuvant chemotherapy [12].

Regarding treatment strategies, interventions like cognitive behavioral therapy and supportive‐expressive group therapy have proven to have a positive effect on breast cancer survivors [13]. Concurrently, exercise, as a nondrug intervention, has been effective in alleviating symptoms of depression and anxiety in breast cancer survivors, though its precise effect on these psychological symptoms warrants further investigation. Exercise has been found to improve mental health by mitigating symptoms of depression and anxiety in both clinical and nonclinical populations [14, 15]. Blumenthal et al. [16] discovered that exercise is an effective first‐line treatment for mild‐to‐moderate depression, yielding superior results compared to antidepressant medication. Furthermore, Philippot et al. [17] and Gordon et al. [18] found that regular physical activity significantly reduced symptoms of depression and anxiety in adolescents.

In recent years, research has commenced exploring the impact of exercise on the mental health of breast cancer survivors, albeit with results exhibiting some inconsistencies. Liu et al. [19] conducted an 8‐week yoga intervention study and found that yoga can effectively alleviate depression and anxiety symptoms. Similarly, Shehab et al. [20] implemented a 4‐week exercise intervention with 72 breast cancer survivors and found that the intervention positively influenced depression, anxiety, and quality of life. However, Prystupa et al. [21] observed that a mixed exercise intervention improved anxiety symptoms but did not significantly affect depression symptoms. Zhang et al. [22] and Desautels et al. [23] noted that exercise had a positive effect on depression symptoms in breast cancer survivors but had a minimal effect on anxiety symptoms. In addition, Gokal et al. [24] demonstrated that exercise positively influenced fatigue, self‐esteem, mood, physical activity levels, and motivation readiness stages, yet did not significantly affect depression and anxiety. These discrepancies underscore the uncertainty in determining the most efficacious exercise regimen (encompassing type, frequency, and duration) for reducing depression and anxiety in breast cancer survivors.

Multiple meta‐analyses have examined the effects of exercise interventions on depression or anxiety in breast cancer patients from various perspectives. For instance, Sun et al. [25] explored the influence of exercise on depression and anxiety in breast cancer patients, but their study only included those who had completed initial treatments, excluding patients undergoing ongoing treatment or those with recurrent or metastatic disease. Therefore, the findings may not be applicable to breast cancer patients at other stages of the disease. Additionally, Patsou et al. [26] conducted a meta‐analysis exclusively focusing on studies published after 2011, overlooking earlier relevant research, which may have contributed to discrepancies in their conclusions. Besides, Salam et al. [27] examined the effects of exercise on depressive symptoms in breast cancer patients, using scapular functional training as an exercise intervention. Whether functional joint training can be utilized as an exercise intervention remains to be proven. In the meta‐analysis conducted by Zhu et al. [28] and Ramírez‐Vélez et al. [29], some studies included a control group that performed stretching and flexibility exercises, making it more challenging to differentiate the effects of exercise interventions. Furthermore, the meta‐analyses conducted by Lee et al. [30] and Sikandari et al. [31] focused on single outcome measures, such as the Hospital Anxiety and Depression Scale (HADS) or the Epidemiological Studies Depression Scale (CES‐D). Due to the diverse range of scales used to assess depression and anxiety, the conclusions of these studies may have certain limitations. Regarding the specifics of exercise interventions, both Ramírez‐Vélez et al. [29] and Patsou et al. [26] discussed the total weekly duration of the exercise interventions for breast cancer patients but did not provide detailed information about the specific structure of the interventions, such as the duration of each session or the frequency of sessions per week.

Therefore, this meta‐analysis is grounded in rigorous randomized controlled trials (RCTs) that aim to investigate the effects of exercise on depression and anxiety in breast cancer survivors and to ascertain the optimal exercise regimen for this patient population.

## 2 Materials and Methods

### 2.1 Design

The Cochrane Selection Manual and the Preferred Reporting Items for Systematic Reviews and Meta‐Analysis (PRISMA, 2020) guidelines [32] were followed for conducting this systematic review and meta‐analysis. The protocol was registered on PROSPERO, with the registration number CRD42024524751.

### 2.2 Search Strategy

For this study, literature searches were conducted up to October 28, 2023, across five major databases: PubMed, Web of Science, Cochrane, Scopus, and Embase. The search process used keywords and Medical Subject Headings (MESH) such as exercise, cancer, depression, and anxiety to locate all exercise‐related studies on depression and anxiety in breast cancer survivors. In addition, to augment the search results, the reference lists of selected studies were manually scrutinized to identify potentially eligible articles. Two authors (Y. Z. and G. L.) independently searched and screened the studies based on predetermined criteria. In cases of disagreement, a third author (L. Y.) was consulted until a consensus was reached.

### 2.3 Eligibility Criteria

Inclusion criteria encompassed: (1) studies designed as RCTs; (2) inclusion of both an intervention and a control group; (3) utilization of breast cancer survivors as participants; and (4) measurement of depression and anxiety as outcomes.

Exclusion criteria were as follows: (1) non‐English publications; (2) reviews and conference articles; (3) studies involving animal models; (4) publications with a high risk of bias or lacking full‐text access; and (5) outcome indicators that could not be converted into mean and standard deviation (SD) values.

### 2.4 Data Extraction

Two authors (Y. Z. and G. L.) independently extracted the data, comprising: (1) study characteristics, including the first author's surname, publication year, and sample size; (2) intervention details, such as type, duration, frequency, total treatment duration, and intervention duration per week; (3) participant characteristics, encompassing age, body mass index (BMI), and time since diagnosis; and (4) efficacy outcomes, specifically the mean and SD of depression and anxiety score changes from baseline to postintervention.

### 2.5 Methodological Quality Assessment

The assessment of the risk of bias was independently conducted by two authors (Y. Z. and G. L.), and any discrepancies were resolved through discussion. The Cochrane Randomized Trials Risk of Bias Tool (RoB‐2) was utilized for this assessment [33], which examines six domains: randomization sequence generation, allocation concealment, blinding, incomplete outcome data, selection of reported results, and other biases. Each domain was assigned a risk level of “low,” “high,” or “unclear.” [34].

### 2.6 Statistical Analysis

Since depression and anxiety were assessed using diverse questionnaires, data were analyzed using a random‐effects model to derive standardized mean difference (SMD) and 95% confidence interval (CI). For studies reporting standard errors (SE) or 95% CIs, the corresponding SD was estimated [35, 36]. In addition, the analysis of image data was performed using the online software PlotDigitizer [37, 38]. In cases where significant heterogeneity (*I* ^2^ > 50%) was detected, subgroup and sensitivity analyses were conducted to explore the sources of heterogeneity.

In subgroup analyses, the effect of exercise on depression and anxiety was assessed based on type (aerobic exercise, resistance exercise, and multicomponent training [a training modality that involves different physical capacities in the same exercise session] [39]), session duration (≤ 60 and > 60 min), and frequency (< 3 and ≥ 3 times per week). The forest plot was generated using RevMan.5, while Egger's test, sensitivity analysis, and funnel plot were performed using Stata 18. The results were considered statistically significant if the *p* value was < 0.05.

## 3 Results

### 3.1 Study Selection

**FIGURE 1:**
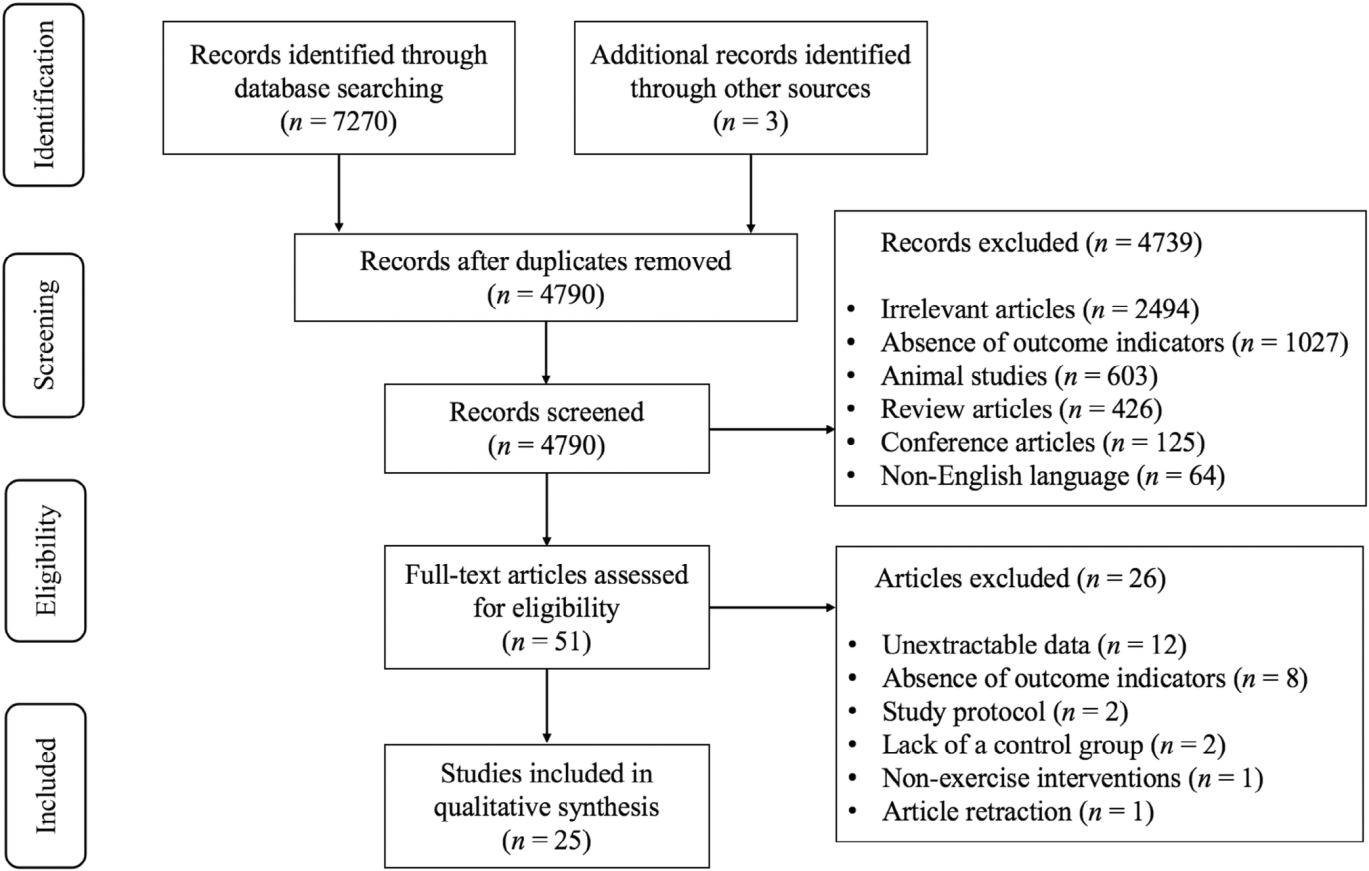
PRISMA flowchart of study selection.

As shown in Figure 1, 7270 article records were initially identified from the databases and 3 records from other sources. After excluding duplicates, 4790 studies remained. Screening of titles and abstracts resulted in the exclusion of 4739 studies that did not meet the inclusion criteria. Upon full‐text reading of the remaining 51 studies, 26 were excluded for the following reasons: (1) unextractable data (*n* = 12); (2) absence of outcome indicators (*n* = 8); (3) study protocol (*n* = 2); (4) lack of a control group (*n* = 2); (5) nonexercise interventions (*n* = 1); and (6) article retraction (*n* = 1). Finally, 25 studies [20, 21, 22, 24, 40, 41, 42, 43, 44, 45, 46, 47, 48, 49, 50, 51, 52, 53, 54, 55, 56, 57, 58, 59, 60] were deemed eligible for systematic review and meta‐analysis.

### 3.2 Characteristics of the Included Studies

Table 1 outlines the primary characteristics of the included studies. Of the studies, four were conducted in China [22, 41, 43, 51] and the United States [42, 47, 50, 57], three in South Korea [49, 52, 53] and Germany [45, 48, 58], two in Canada [44, 54] and Iraq [20, 46], and one in the United Kingdom [24], France [55], Ukraine [21], India [40], Netherlands [56], Iran [59], and Spain [60]. All included studies comprised 2577 female participants diagnosed with breast cancer. The intervention and control groups consisted of 1308 and 1269 participants, respectively. The 25 studies were published between 2007 and 2023. Among them, 14 studies involved multicomponent training [21, 22, 42, 46, 48, 49, 50, 52, 53, 55, 56, 57, 59, 60], 10 focused on aerobic exercise [20, 24, 40, 41, 43, 44, 45, 51, 54, 58], and 2 included resistance exercise [44, 54], with 1 study not specifying the intervention type [47]. The specific intervention varied, encompassing walking, Tai Chi, Qigong, Baduanjin, yoga, cycling, sit‐ups, weight lifting, muscle stretching, and dancing. The intervention duration ranged from 3 to 26 weeks, averaging 11.92 weeks, except for one study that did not report the duration [57]. The frequency of interventions varied from 1 to 6 times per week, averaging 3.05 times per week, with two studies omitting this information [44, 49]. Session duration lasted between 30 and 125 min, averaging 61.67 min, with four studies not detailing session durations [44, 47, 49, 54]. The minimum weekly time was 60 min, while the average weekly time amounted to 167.72 min. However, four studies [44, 47, 49, 54] failed to report the specific weekly time.

**TABLE 1:** Characteristics of the studies included in this meta‐analysis.

Study	Country	Sample size	Age (years)	BMI	Type of intervention	Intervention	Duration (Weeks)	Frequency (Times/week)	Minutes per session (min)	Intensity	Supervision	Depression outcomes	Anxiety outcomes
Cadmus et al. [42]‐YES	United States	IG: 37 CG: 37	IG: 54.5 ± 8.2 CG: 54.0 ± 10.9	IG: 27.9 ± 5.3 CG: 27.5 ± 5.4	Multicomponent	PA, RA, WA	24	5	30	Moderate‐high	No	CES‐D	STAI
Cadmus et al. [42]‐IMPACT	United States	IG: 25 CG: 25	IG: 56.5 ± 9.5 CG: 55.1 ± 7.7	IG: 30.4 ± 6.0 CG: 30.1 ± 7.4	Multicomponent	PA, RA, WA	24	5	30	Moderate‐high	Yes	CES‐D	STAI
Carayol et al. [55]	France	IG: 72 CG: 63	52 ± 10	25.5 ± 5.3	Multicomponent	AE, RE	26	3	50	Moderate	First nine sessions supervised	HADS	HADS
Charati et al. [59]	Iran	IG: 35 CG: 35	IG: 38.14 ± 10.70 CG: 42.63 ± 8.11	NA	Multicomponent	WA, Stretching	5	3–5↑	20–30↑	NA	Yes	HADS	HADS
Courneya et al. [44]‐AET‐1	Canada	IG: 78 CG: 82	49.2	26.6 ± 5.5	Aerobic	AE	18	3	55	Moderate‐high	NA	CES‐D	STAI
Courneya et al. [44]‐RET‐1	Canada	IG: 82 CG: 82	49.2	NA	Resistance	RE	17	3	NA	Moderate‐high	NA	CES‐D	STAI
Courneya et al. [44]‐AET‐2	Canada	IG: 78 CG: 82	49	NA	Aerobic	AE	4	3	60	Moderate‐high	NA	CES‐D	STAI
Courneya et al. [44]‐RET‐2	Canada	IG: 82 CG: 82	49	NA	Resistance	RE	4	3	NA	Moderate‐high	NA	CES‐D	STAI
Cramer et al. [58]	Germany	IG: 19 CG: 21	IG: 48.3 ± 4.8 CG: 50.0 ± 6.7	NA	Aerobic	Yoga, Meditation	12	1	90	NA	Yes	HADS	HADS
Gokal et al. [24]	United Kingdom	IG: 25 CG: 25	IG: 52 ± 11.7 CG: 52 ± 8.9	IG: 27.2 ± 4.82 CG: 28.25 ± 5.83	Aerobic	WA	12	5	30	Moderate	NA	HADS	HADS
Ho et al. [43]	Hong Kong, China	IG: 69 CG: 64	IG: 48.6 ± 7.7 CG: 49.1 ± 8.7	NA	Aerobic	DA	3	2	90	NA	Yes	HADS	HADS
Kareem et al. [46]	Iraq	IG: 44 CG: 44	IG: 39.59 ± 7.15 CG: 42.81 ± 6.38	NA	Multicomponent	PMR	8	2	60	NA	Yes	HADS	HADS
Kim et al. [52]	Korea	IG: 23 CG: 22	IG: 44.6 ± 9.9 CG: 47.1 ± 7.3	IG: 22.4 ± 3.2 CG: 22.7 ± 2.5	Multicomponent	SSED	12	5	30	NA	NO	HADS	HADS
Kim et al. [53]	Korea	IG: 23 CG: 25	IG: 49.91 ± 7.62 CG: 48.48 ± 6.75	NA	Multicomponent	AE, ST	12	1–2	120	NA	Yes	KHADS	KHADS
Knoerl et al. [57]	United States	IG: 26 CG: 21	52.8 ± 8.8	30.2 ± 6.5	Multicomponent	AE, ST	NA	2	110	NA	Yes	HADS	HADS
Lee et al. [49]	Korea	IG: 29 CG: 29	IG: 41.5 ± 6.3 CG: 43.2 ± 5.1	NA	Multicomponent	WSEDI	12	2	NA	NA	No	HADS	HADS
Mehnert et al. [45]	Germany	IG: 30 CG: 28	51.88 ± 8.46	NA	Aerobic	ST, MG, WA, JO	10	2	90	NA	Yes	HADS	HADS
Prystupa et al. [21]	Ukraine	IG: 26 CG: 25	IG: 56.24 ± 2.06 CG: 56.80 ± 2.14	IG: 25.82 ± 0.4 CG: 26.0 ± 0.62	Multicomponent	PR	4	5	45	NA	Yes	HADS	HADS
Rao et al. [40]	India	IG: 33 CG: 36	49.2 ± 9.6	NA	Aerobic	Yoga	4	1	60	NA	Yes	STAI	BDI
Rogers et al. [50]	United States	IG: 20 CG: 24	56.2 ± 7.7	IG: 29.8 ± 4.8 CG: 32.6 ± 6.6	Multicomponent	AE, ST	12	4	40	Moderate‐high	Yes	PROMIS	PROMIS
Rogers et al. [47]	United States	IG: 110 CG: 112	54.4 ± 8.5	NA	NA	Personalized training	12	1	NA	NA	Yes	HADS	HADS
Salchow et al. [48]	Germany	IG: 30 CG: 21	IG: 54.23 ± 7.846 CG: 51.52 ± 8.412	NA	Multicomponent	Kyusho Jitsu	24	2	90	NA	Yes	HADS	HADS
Shehab et al. [20]	Iraq	IG: 24 CG: 24	IG: 46.17 ± 3.28 CG: 48.07 ± 3.49	NA	Aerobic	Bike	4	2	30	Low to high↑	Yes	HADS	HADS
Travier et al. [56]	Netherlands	IG: 87 CG: 77	IG: 49.7 ± 8.2 CG: 49.5 ± 7.9	IG: 25.8 ± 4.4 CG: 26.6 ± 5.2	Multicomponent	AE, RE	18	2	60	Moderate‐high	Yes	HADS	HADS
Villanueva et al. [60]	Spain	IG: 32 CG: 35	IG: 49 ± 9 CG: 48 ± 9	NA	Multicomponent	AE, Stretching	8	3	125	NA	Yes	POMS	POMS
Wei et al. [41]	China	IG: 35 CG: 35	60	23.06 ± 2.54	Aerobic	Baduanjin	4	5	40	NA	Yes	HADS	HADS
Ying et al. [51]	China	IG: 46 CG: 40	54.09	IG: 25.04 ± 2.88 CG: 24.72 ± 3.2	Aerobic	Baduanjin	24	3	60	NA	Yes	GAD‐7	PHQ‐9
Zhang et al. [22]	China	IG: 92 CG: 92	IG: 48.58 ± 14.77 CG: 49.27 ± 10.33	NA	Multicomponent	AE, Stretching	5	6	60	NA	Yes	HADS	HADS

Abbreviations: ↑, gradually increase until target time; AE, aerobic training; BDI, the Beck's Depression Inventory; BMI, body mass index; CES‐D, the Epidemiological Studies Depression Scale; CG, control groups; GAD‐7, the Generalized Anxiety Disorder‐7 Scale; HADS, the Hospital Anxiety and Depression Scale; IG, Intervention groups; KHADS, the Korean version of the Hospital Anxiety and Depression Scale; moderate–high, moderate‐ to‐high‐intensity exercise; NA, not available; No, intervention process is not supervised; PA, physical activity; PHQ‐9, the Patient Health Questionnaire‐9; POMS, the Profile of Mood State Questionnaire; PROMIS, the Patient‐Reported Outcomes Measurement Information System; RA, recreational activities; RE, resistance training; ST, strength training; STAI, the State–Trait Anxiety Index; Yes, intervention process is monitored.

### 3.3 Meta‐Analysis

**FIGURE 2:**
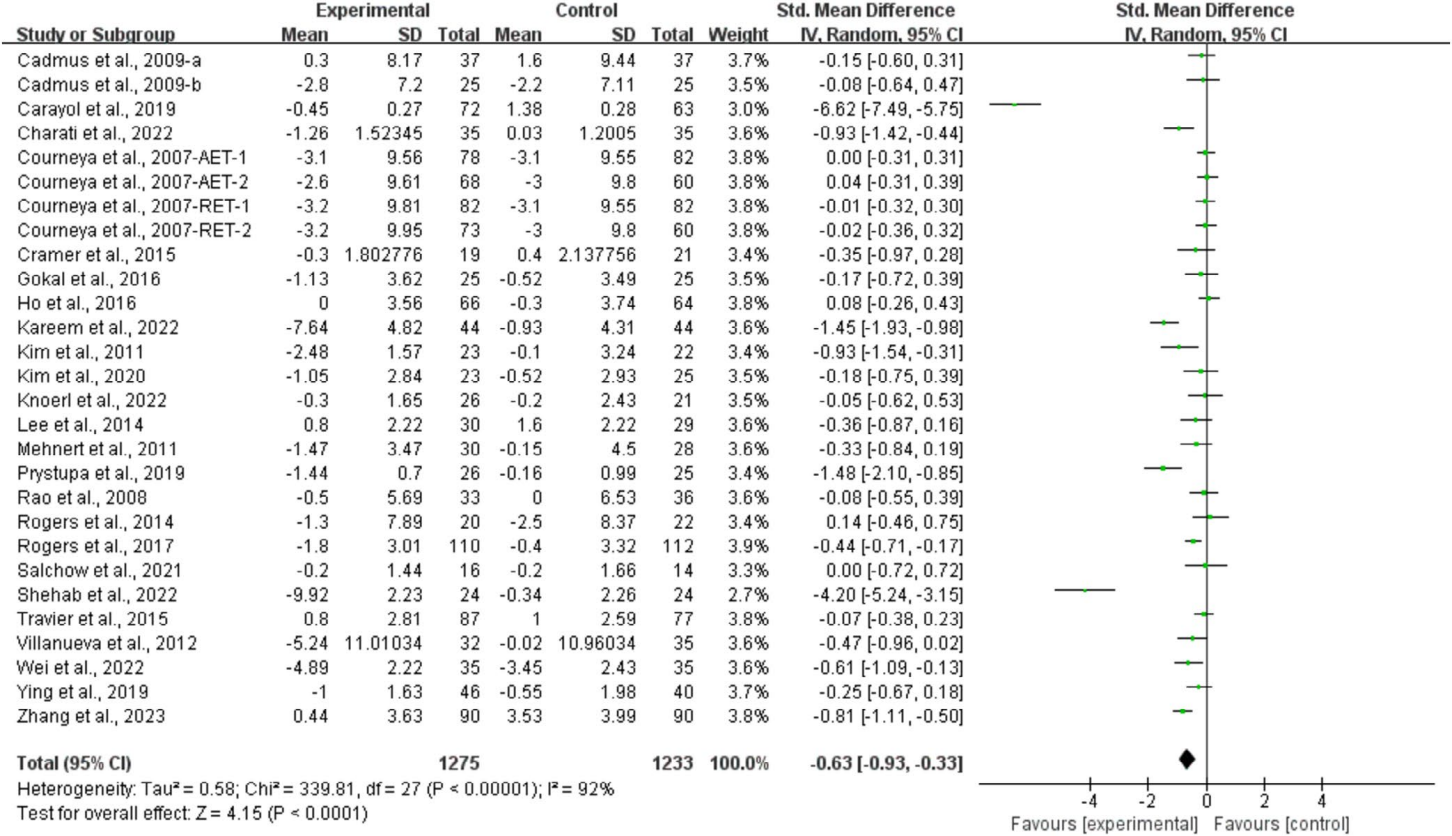
Results of the meta‐analysis of the effects of exercise on depression in breast cancer survivors.

**FIGURE 3:**
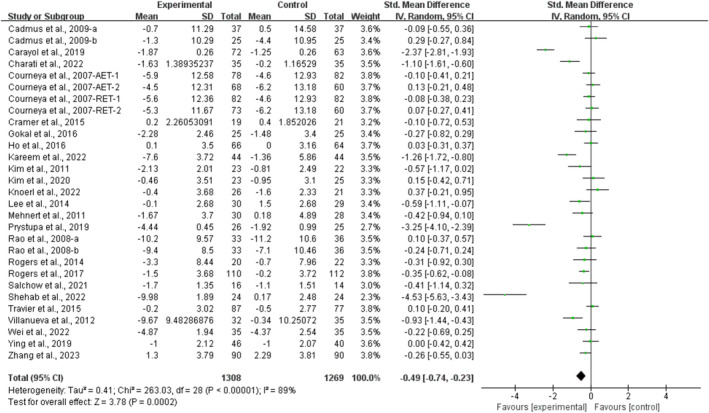
Results of the meta‐analysis of the effects of exercise on anxiety in breast cancer survivors.

Compared with the control group, exercise significantly alleviated depression [SMD, −0.63 (95% CI, −0.93 to −0.33), *p* < 0.0001, *I* ^2^ = 92%, Figure 2] and anxiety [SMD, −0.49 (95% CI, −0.74 to −0.23), *p* = 0.0002, *I* ^2^ = 89%, Figure 3] in breast cancer survivors.

### 3.4 Subgroup Analysis

**FIGURE 4:**
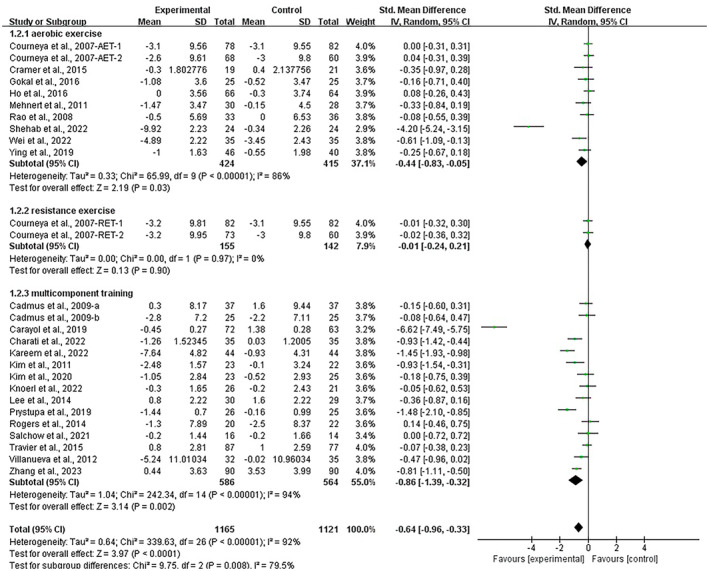
Results of the meta‐analysis on the effects of types of intervention on depression in breast cancer survivors.

Stratifying the analysis by intervention type, aerobic exercise [SMD, −0.44 (95% CI, −0.83 to −0.05), *p* = 0.03, *I* ^2^ = 86%], and multicomponent training had a significant effect on alleviating depression [SMD, −0.86 (95% CI, −1.39 to −0.32), *p* = 0.002, *I* ^2^ = 94%] in breast cancer survivors, with multicomponent training being the most effective intervention. However, resistance exercise showed no significant improvement in depression in breast cancer survivors [SMD, −0.01 (95% CI, −0.24 to 0.21), *I* ^2^ = 0%, *p* = 0.90, *I* ^2^ = 0%, Figure 4].

**FIGURE 5:**
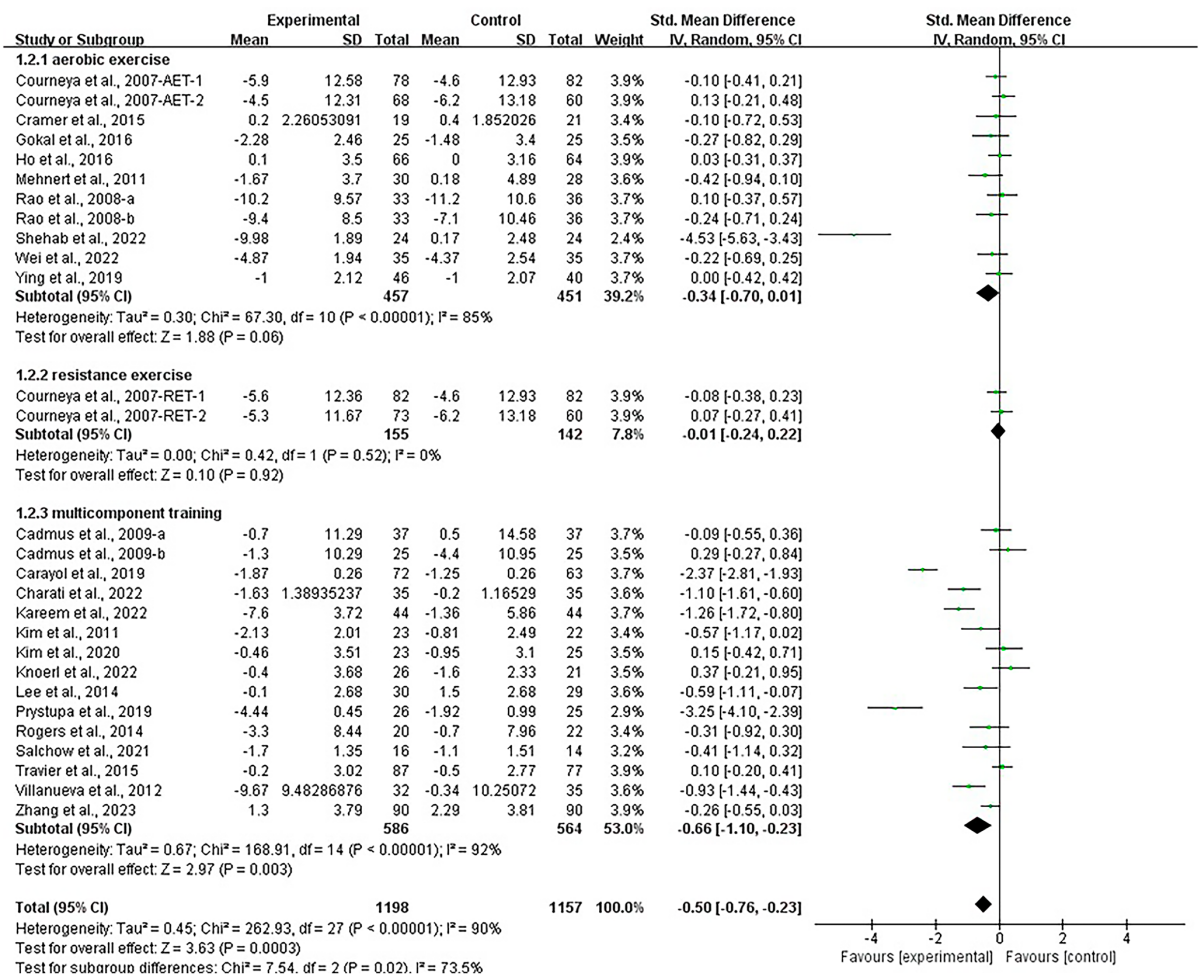
Results of the meta‐analysis on the effects of types of interventions on anxiety in breast cancer survivors.

Concurrently, multicomponent training had a significant effect on alleviating anxiety [SMD, −0.66 (95% CI, −1.10 to −0.23), *p* = 0.003, *I* ^2^ = 92%] in breast cancer survivors. However, aerobic exercise [SMD, −0.34 (95% CI, −0.70 to 0.01), *p* = 0.06, *I* ^2^ = 85%] and resistance exercise [SMD, −0.01 (95% CI, −0.24 to 0.22), *p* = 0.92, *I* ^2^ = 0%, Figure 5] showed no significant improvement in depression in breast cancer survivors.

Since multicomponent training was the most effective intervention type in alleviating depression and anxiety, we conducted further subgroup analyses focusing on multicomponent training.

#### 3.4.1 Depression

**FIGURE 6:**
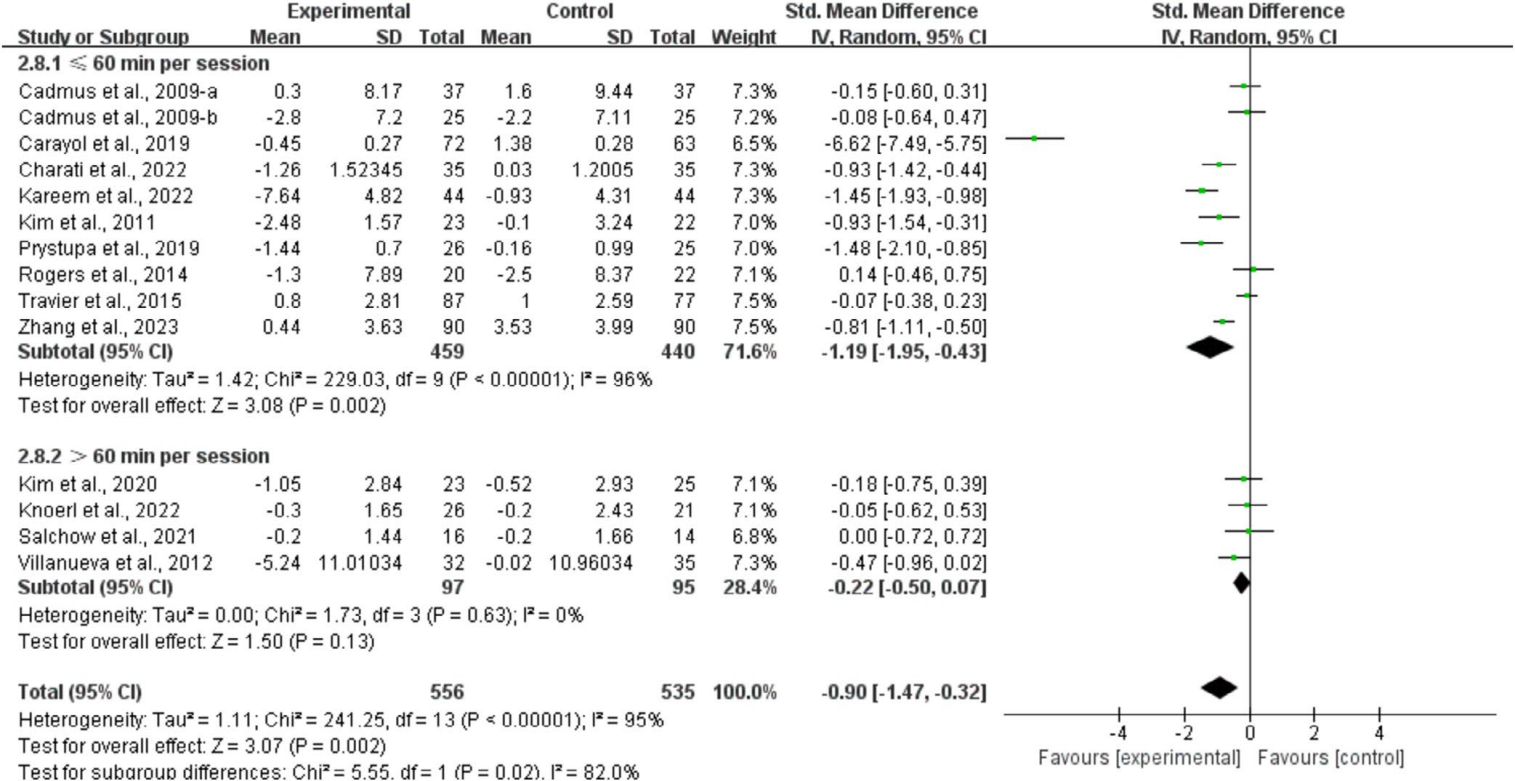
Results of the meta‐analysis of the effects of the duration of multicomponent training per session on depression in breast cancer survivors.

Stratifying the analysis by session duration, multicomponent training conducted for ≤ 60 min per session significantly alleviated depression in breast cancer survivors [SMD, −1.19 (95% CI, −1.95 to −0.43), *p* = 0.002, *I* ^2^ = 96%]. However, multicomponent training conducted for > 60 min per session had no significant association with depression in breast cancer survivors [SMD, −0.22 (95% CI, −0.50 to 0.07), *p* = 0.13, *I* ^2^ = 0%, Figure 6].

**FIGURE 7:**
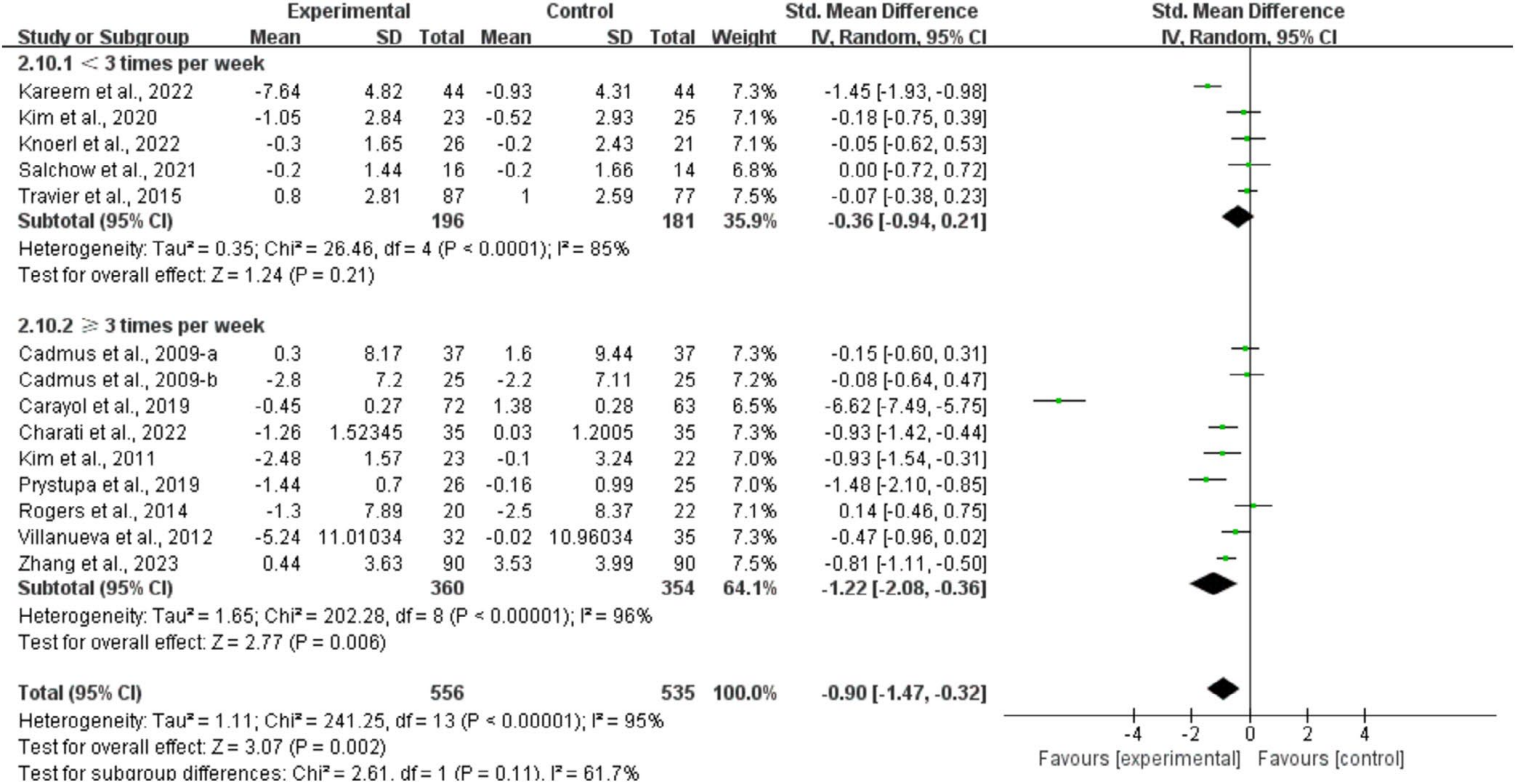
Results of a meta‐analysis of the effects of the frequency of multicomponent training on depression in breast cancer survivors.

Additionally, when analyzing the subgroups by frequency, multicomponent training conducted for ≥ 3 times per week significantly alleviated depression in breast cancer survivors [SMD, −1.22 (95% CI, −2.08 to −0.36), *p* = 0.006, *I* ^2^ = 96%]. However, multicomponent training conducted for < 3 times per week had no significant association with depression in breast cancer survivors [SMD, −0.36 (95% CI, −0.94 to 0.21), *p* = 0.21, *I* ^2^ = 85%, Figure 7].

#### 3.4.2 Anxiety

**FIGURE 8:**
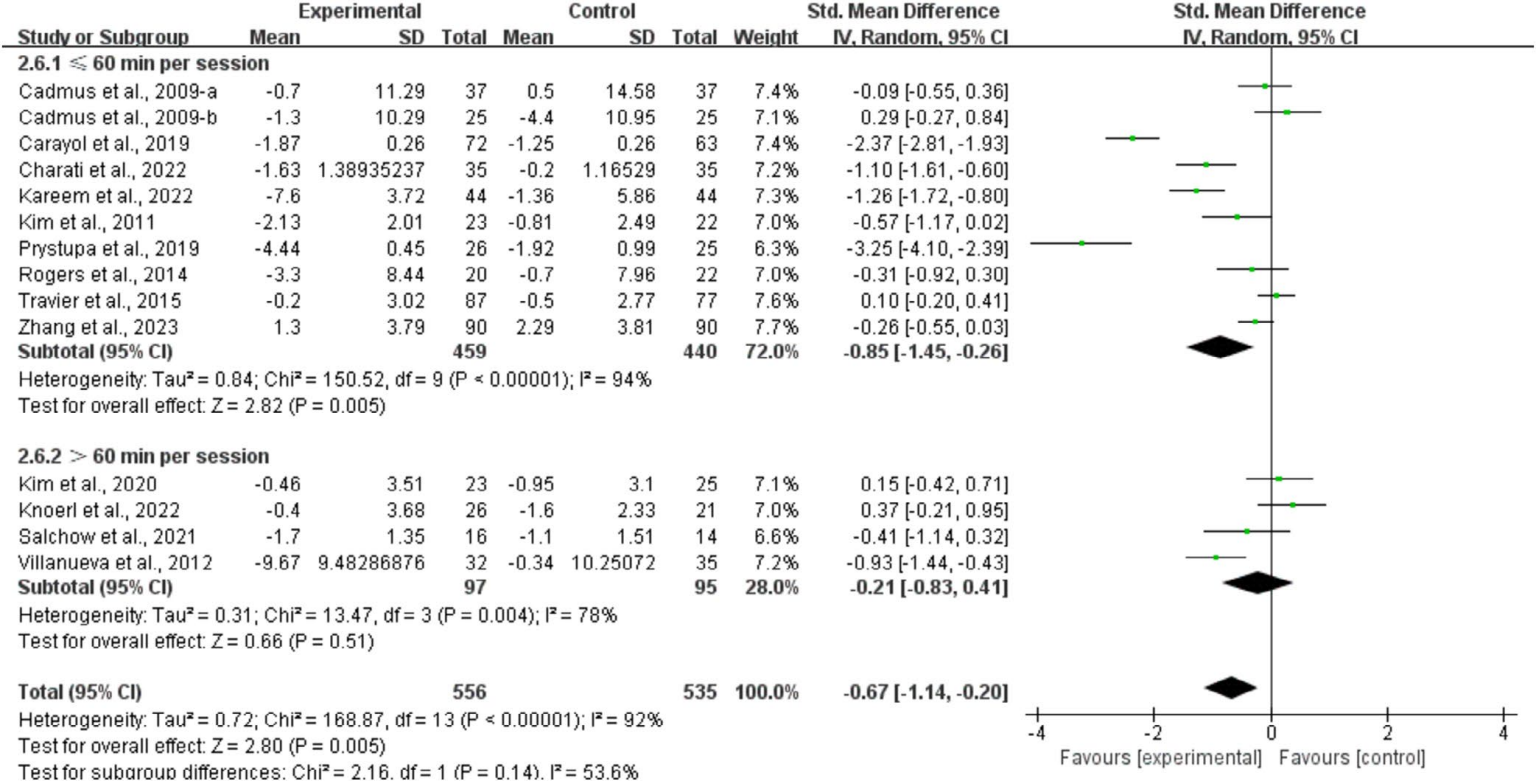
Results of the meta‐analysis of the effects of the duration of multicomponent training per session on anxiety in breast cancer survivors.

Stratifying the analysis by session duration, multicomponent training conducted for ≤ 60 min significantly alleviated anxiety in breast cancer survivors [SMD, −0.85 (95% CI, −1.45 to −0.26), *p* = 0.005, *I* ^2^ = 94%]. However, multicomponent training conducted for > 60 min per session had no significant association with anxiety in breast cancer survivors [SMD, −0.21 (95% CI, −0.83 to 0.41), *p* = 0.51, *I* ^2^ = 78%, Figure 8].

**FIGURE 9:**
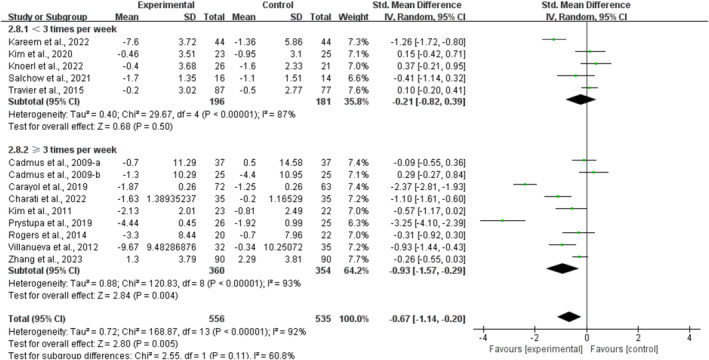
Results of a meta‐analysis of the effects of frequency of multicomponent training on anxiety in breast cancer survivors.

Furthermore, when analyzing the subgroups by frequency, multicomponent training conducted for ≥ 3 times per week significantly alleviated anxiety in breast cancer survivors [SMD, −0.93 (95% CI, −1.57 to −0.29), *p* = 0.004, *I* ^2^ = 93%]. However, multicomponent training conducted for < 3 times per week had no significant association with anxiety in breast cancer survivors [SMD, −0.21 (95% CI, −0.82 to 0.39), *p* = 0.50, *I* ^2^ = 87%, Figure 9].

### 3.5 Adverse Events

Adverse events related to exercise intervention were reported in 2 of 25 studies (8.0%). A total of 16 adverse events were documented. Specifically, eight adverse events occurred in the intervention group and eight adverse events occurred in the control group. Furthermore, four adverse events were possibly related to yoga practice, while the majority of adverse events were not associated with the exercise intervention, suggesting that exercise was safe for breast cancer patients.

### 3.6 Risk of bias

The Cochrane Randomized Trials Risk of Bias Tool (RoB‐2) tool was utilized to evaluate the risk of bias in the included studies, considering factors such as selection, performance, detection, attrition, reporting, and other biases. As illustrated in Figure S1, the overall quality of the studies was categorized into three levels: low, moderate, and high. Specifically, 3 trials posed a low risk of bias, 24 trials presented a moderate risk, and 2 trials exhibited a high risk of bias.

### 3.7 Publication bias

The funnel plot was used to detect publication bias in all 28 depression trials. Visual inspection of the funnel plot (Figure S2), along with Egger's test (*t* = −2.88, *p* = 0.008, Table S1), suggested the presence of publication bias due to the asymmetry of the funnel plot. The raw combined effect of exercise on depression, as determined by the trim‐and‐fill method, was −0.631. Upon correcting for publication bias using the trim‐and‐fill method, the overall effect size was −0.640 (95% CI, −0.932 to −0.349, *p* < 0.0001). Despite some publication bias in the results, the conclusions drawn from the publication bias analysis do not undermine the effectiveness of exercise in alleviating depression in breast cancer survivors.

Similarly, the funnel plot was used to detect publication bias in all 29 anxiety trials. Visual examination of the funnel plot (Figure S3) and the results of Egger's test (*t* = −2.60, *p* = 0.015, Table S2) indicated the existence of publication bias, evident in the asymmetry of the funnel plot. The raw overall effect of exercise on anxiety, as per the trim‐and‐fill method, was −0.486. After correcting for publication bias using the trim‐and‐fill method, the overall effect size was −0.503 (95% CI, −0.751 to −0.256, *p* < 0.0001). Despite the presence of some publication bias, the conclusions drawn from the publication bias analysis do not suggest that exercise is ineffective in alleviating anxiety in breast cancer survivors.

### 3.8 Sensitivity Analyses

The sensitivity analysis revealed that regardless of the exclusion of any individual study, the positive effect of exercise on depression (Figure S4) and anxiety (Figure S5) in breast cancer survivors remained stable and consistent in both direction and magnitude.

## 4 Discussion

### 4.1 Main Findings

The aim of this study was to investigate the effect of exercise on depression and anxiety in breast cancer survivors and to ascertain the optimal exercise regimen for this patient population. Out of the initially identified 4790 search records, 25 studies were deemed eligible for systematic review and meta‐analysis. Our results revealed that exercise significantly alleviated depression and anxiety in breast cancer survivors, with multicomponent training being the most effective intervention type. Subgroup analysis further indicated that multicomponent training sessions lasting ≤ 60 min, performed ≥ 3 times per week, significantly alleviated depression and anxiety in breast cancer survivors.

### 4.2 The Effects of Multicomponent Training on Depression in Breast Cancer Survivors

This systematic review and meta‐analysis demonstrated that exercise significantly alleviates depression in breast cancer survivors, as evidenced by an overall reduction in depressive symptom scores of 0.63 (SMD). Additionally, a meta‐analysis conducted by Pérez‐Bilbao et al. [61] concurred, showing that exercise and dietary interventions can effectively diminish depression in breast cancer survivors, aligning with our findings.

Regarding intervention type, aerobic exercise and multicomponent training significantly alleviated depression in breast cancer survivors, with multicomponent training being the most effective type, which holds great clinical significance for breast cancer survivors. Brown et al. [62] supported the efficacy of multicomponent training for cancer survivors, indicating that a combination of diverse training types more effectively improved depressive symptoms. In addition, Campbell et al. [63] showed that resistance training alone was not significantly effective in improving depressive symptoms in breast cancer survivors, aligning with the findings of this study. Furthermore, Zhou et al. [34] showed that aerobic exercise, resistance exercise, and combined training all significantly improved fatigue in breast cancer survivors, with combined training yielding the most therapeutic effect, reinforcing the notion that multicomponent training is the optimal intervention for alleviating symptoms in breast cancer survivors.

However, contrary to the results of this study, some research suggests that resistance exercise may positively affect breast cancer survivors' symptoms. Considering the limited number and poor quality of articles on resistance exercise included in this study, we believe that there is a high possibility that resistance exercise positively affects depressive symptoms in breast cancer survivors. Additionally, given the low exercise tolerance in breast cancer survivors, resistance exercise may exacerbate pain and discomfort, thereby compromising its positive effects.

Our results showed that multicomponent training appears to be the optimal exercise modality for alleviating depression in breast cancer survivors [62, 64]. Multicomponent training is perceived to bolster physical fitness and more effectively address depression, anxiety, fatigue, and sleep disturbances compared to other forms of exercise [65]. Although resistance exercise necessitates a higher fitness level than aerobic exercise and may be less straightforward to implement, its high intensity predisposes it to foster greater exercise adaptation. Therefore, aerobic exercise was prioritized initially, and upon enhancing physical fitness, resistance exercise was incorporated to constitute a multicomponent training regimen.

Exclusively multicomponent training studies were analyzed in subgroups within this study, as it exhibits superior intervention outcomes. The objective was to furnish theoretical grounding for the design of multicomponent training interventions. Notably, multicomponent training significantly alleviated depression in breast cancer survivors. However, the study also identified substantial heterogeneity. Therefore, subgroup analyses were conducted to elucidate the results. These analyses unveiled that multicomponent training lasting ≤ 60 min per session and ≥ 3 times per week markedly diminished depression in breast cancer survivors.

In terms of intervention frequency, multicomponent training conducted ≥ 3 times per week significantly reduced depression in breast cancer survivors, whereas training < 3 times per week had no notable impact, which aligns with the findings of Campbell et al. [63], showing that engaging in at least 3 moderate‐intensity workouts per week significantly improved depression in cancer survivors. In addition, Craft et al. [66] found that five workouts per week were more effective in alleviating depression than two to four workouts. The American College of Sports Medicine (ACSM) guidelines recommend five low‐ to moderate‐intensity workouts per week for optimal training outcomes [67]. An intervention frequency of at least three times per week exerts a marked influence on depression, likely due to fostering a habitual exercise routine [68]. Furthermore, there appears to be a dose–response relationship between exercise volume and depression reduction, indicating that increased exercise amounts may correlate with enhanced symptom relief [63]. Zhou et al. [34] concurred, stating that the optimal training effect occurred with an intervention frequency of at least three times per week and a total of at least 180 min per week. It is also recommended that breast cancer survivors shorten individual intervention durations and increase frequencies to achieve better therapeutic effects. However, our study did not uncover a correlation between improved physical function and exercise dosage in breast cancer survivors, suggesting that increasing exercise frequency may yield a stronger cumulative effect, thereby optimizing training outcomes, improving physical function, and mitigating depression.

Regarding intervention duration, sessions lasting ≤ 60 min significantly reduced depression in breast cancer survivors, whereas longer sessions had no significant effect. The findings of Rethorst et al. [69] support this, showing that exercise sessions lasting 45 to 59 min had a stronger antidepressant effect. Similarly, Li et al. [39] found no significant improvement in cognitive function in multiple sclerosis patients with exercise interventions exceeding 60 min, implying that prolonged exercise may not yield additional benefits. Given that breast cancer survivors often have lower exercise tolerance than healthy individuals, extended exercise sessions may induce fatigue and exacerbate depressive mood. Moreover, breast cancer survivors frequently experience discomfort and pain during exercise, further diminishing their exercise tolerance [47]. Therefore, enhancing exercise frequency rather than merely extending exercise durations may be a more effective approach.

### 4.3 The Effects of Multicomponent Training on Anxiety in Breast Cancer Survivors

This systematic review and meta‐analysis demonstrated that exercise can significantly alleviate anxiety in breast cancer survivors, leading to an overall reduction in anxiety scores by 0.49 (SMD). A study conducted by Loh et al. [70] indicated that a structured exercise program spanning 6 weeks significantly improved anxiety and emotional distress in elderly cancer survivors. In addition, a retrospective study showed that exercise positively alleviated anxiety, thereby improving both overall and disease‐specific health‐related quality of life (HRQoL) in cancer survivors [71].

The current study investigated the effect of exercise on anxiety in breast cancer survivors and yielded inconsistent findings. While several studies indicated no significant effect of exercise on alleviating anxiety in cancer survivors [22, 23, 72], the majority of studies have established that exercise can significantly alleviate anxiety in breast cancer survivors. The discrepancy might be attributed to the relatively low baseline anxiety of cancer survivors at the commencement of these studies.

From an intervention model perspective, the present study discovered that only multicomponent training led to a significant reduction in anxiety in breast cancer survivors. While certain studies have indicated that aerobic exercise and resistance exercise might significantly lower anxiety in breast cancer survivors, stronger evidence favors the implementation of multicomponent training for alleviating anxiety in breast cancer survivors [62, 63, 73]. However, there is a lack of studies comparing the relative efficacy of aerobic exercise, resistance exercise, and multicomponent training in treating anxiety in breast cancer survivors. Multicomponent training is considered more potent in alleviating anxiety, depression, fatigue, and sleep disturbances due to its more comprehensive physical adaptations [74]. Furthermore, its higher training volume and intensity are more likely to trigger these adaptations.

Given multicomponent training's proven effectiveness in alleviating anxiety in breast cancer survivors, this study specifically conducted a subgroup analysis focusing on this approach. The analysis showed that multicomponent training significantly alleviated anxiety in breast cancer survivors, albeit with notable variations between studies. The subgroup analysis further identified that multicomponent training sessions lasting ≤ 60 min and conducted ≥ 3 times per week led to a significant reduction in anxiety in breast cancer survivors.

Previous research reported that aerobic exercise conducted at least three times per week can effectively alleviate anxiety in cancer survivors and that the effect is even more pronounced when training is undertaken five times per week compared to two to four times per week, thereby reinforcing our perspective [63, 69, 73]. Due to the increased weekly exercise volume, a frequency of exercising more than three times per week is hypothesized to be more conducive to fostering physical adaptation. In addition, an intervention frequency of at least three times per week may foster a habit of regular exercise, which subsequently has a favorable influence on the amelioration of anxiety. Regarding intervention frequencies of less than three times per week, Zhou et al. [34] found that they can still significantly improve fatigue in breast cancer survivors. Therefore, it is imperative to acknowledge the potential benefits of intervention frequencies below three times per week, and such frequencies can still be considered when considering the intervention's duration and measures.

In terms of the duration of a single intervention, sessions lasting ≤ 60 min have been found to significantly diminish anxiety in breast cancer survivors, whereas longer interventions exceeding 60 min are ineffective. Given the low exercise tolerance of breast cancer survivors, we posit that the primary reason for this phenomenon is that extended exercise may induce fatigue, which not only exacerbates the physical strain on breast cancer survivors but may also augment anxiety, thereby diminishing the positive mental health benefit of exercise [34]. Furthermore, considering the variations in physical capabilities in breast cancer survivors, prolonged interventions may surpass their physical limits, leading to discomfort and pain, which can adversely impact mental health [47]. Moreover, prolonged exercise can lead to boredom and a loss of motivation, particularly in the absence of adequate motivation and support. This lack of perseverance may further undermine the effectiveness of exercise in alleviating anxiety.

### 4.4 Limitations

There are several limitations to this study that ought not to be overlooked. Firstly, the included studies were all RCTs of exercise interventions, where complete blinding was unfeasible. Therefore, subjective factors may have introduced a degree of bias into the quality assessment process. Secondly, some included studies failed to report the exercise intensity in detail, hindering a comprehensive evaluation of the impact of intensity on depression and anxiety in breast cancer survivors. Thirdly, while the exercise interventions in the included studies were relatively uniform in type, there was considerable variation in intervention details, which could be the primary contribution to the high heterogeneity observed in the study results. Additionally, statistical analyses pertaining to exercise intensity and intervention supervision have yet to be conducted, necessitating future studies with larger sample sizes and higher research standards to validate our findings. Furthermore, given the high heterogeneity among the studies, the results should be interpreted with caution and moderation. Moreover, the optimal arrangement of multicomponent training content remains unclear, and further studies are needed to explore the design of exercise prescriptions. Finally, due to the variations in treatment cycles among patients included in different studies, as well as differences in patients' health conditions and factors contributing to anxiety and depression, the results of our study should be interpreted with caution.

## 5 Conclusion

Exercise significantly alleviated depression and anxiety in breast cancer survivors, with multicomponent training being the most effective intervention type. This meta‐analysis provides clinicians with evidence to recommend that breast cancer survivors engage in multicomponent training more than three times per week, with each session lasting no more than 60 min, to alleviate depression and anxiety.

## Author Contributions

**Yifan Zhang:** conceptualization, methodology, data curation, formal analysis, software, investigation, and writing – original draft preparation. **Gen Li:** methodology, data curation, formal analysis, validation, and writing – review and editing. **Shiyan Zhang:** methodology, software, data curation, and writing – review and editing. **Yilun Zhou:** methodology, validation, and writing – review and editing. **Yuanyuan Lv:** software, validation, and writing – review and editing. **Lin Feng:** data curation, visualization, supervision, and writing – review and editing. **Laikang Yu:** conceptualization, resources, supervision, project administration, funding acquisition, and writing – review and editing.

## Conflicts of Interest

The authors declare no conflicts of interest.

## Supporting information


Data S1.

## Data Availability

Data sharing is not applicable to this article as no new data were created or analyzed in this study.
