# Supplementary material for: Effects of Exercise on Depression and Anxiety in Breast Cancer Survivors: A Systematic Review and Meta‐Analysis of Randomized Controlled Trials
Source: Cancer Med. 2025 Mar 7;14(5):e70671. doi: 10.1002/cam4.70671 (PMC11886893; doi:10.1002/cam4.70671)
Supplement: Supplementary file 1 — Data S1. [file CAM4-14-e70671-s001.docx]

**Supplemental material**

**Effects of exercise on depression and anxiety in breast cancer survivors: a systematic review and meta-analysis of randomized controlled trials**

**Figure S1.** Results of Cochrane risk of bias tool.......................................2

**Figure S2.** Funnel plot (depression)..........................................................3

**Figure S3.** Funnel plot (anxiety)................................................................4

**Figure S4.** Sensitivity analysis results (depression)..................................5

**Figure S5.** Sensitivity analysis results (anxiety)........................................6

**Table S1.** Results of Egger’s test (depression)...........................................7

**Table S2.** Results of Egger’s test (anxiety)................................................8

**Figure S1.** Results of Cochrane risk of bias tool


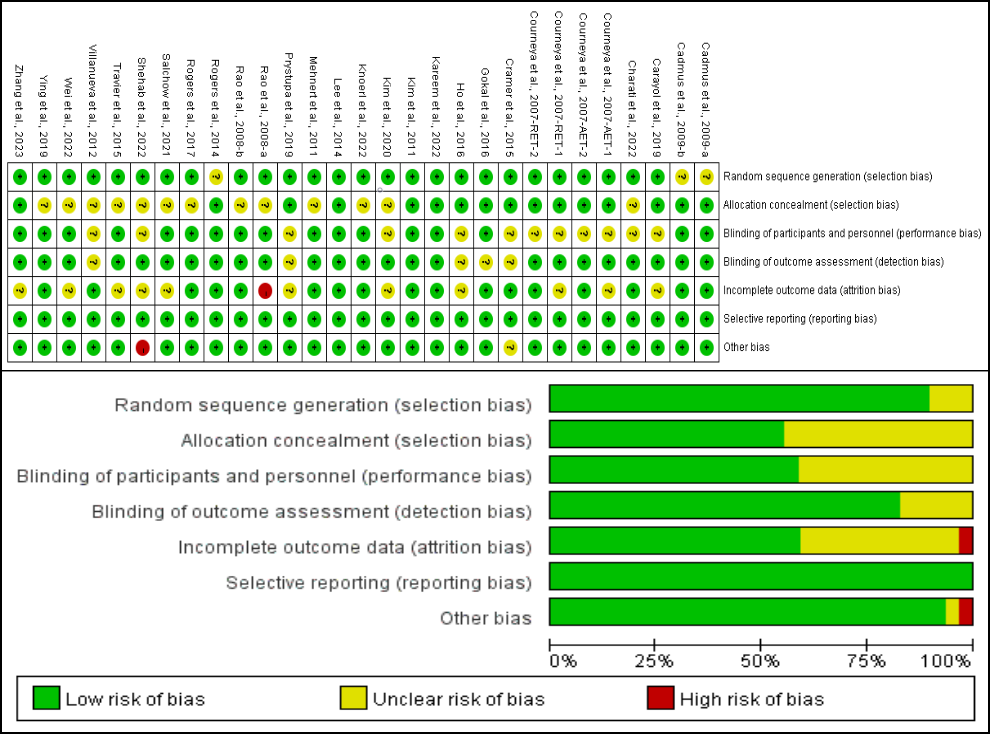


**Figure S2.** Funnel plot (depression)


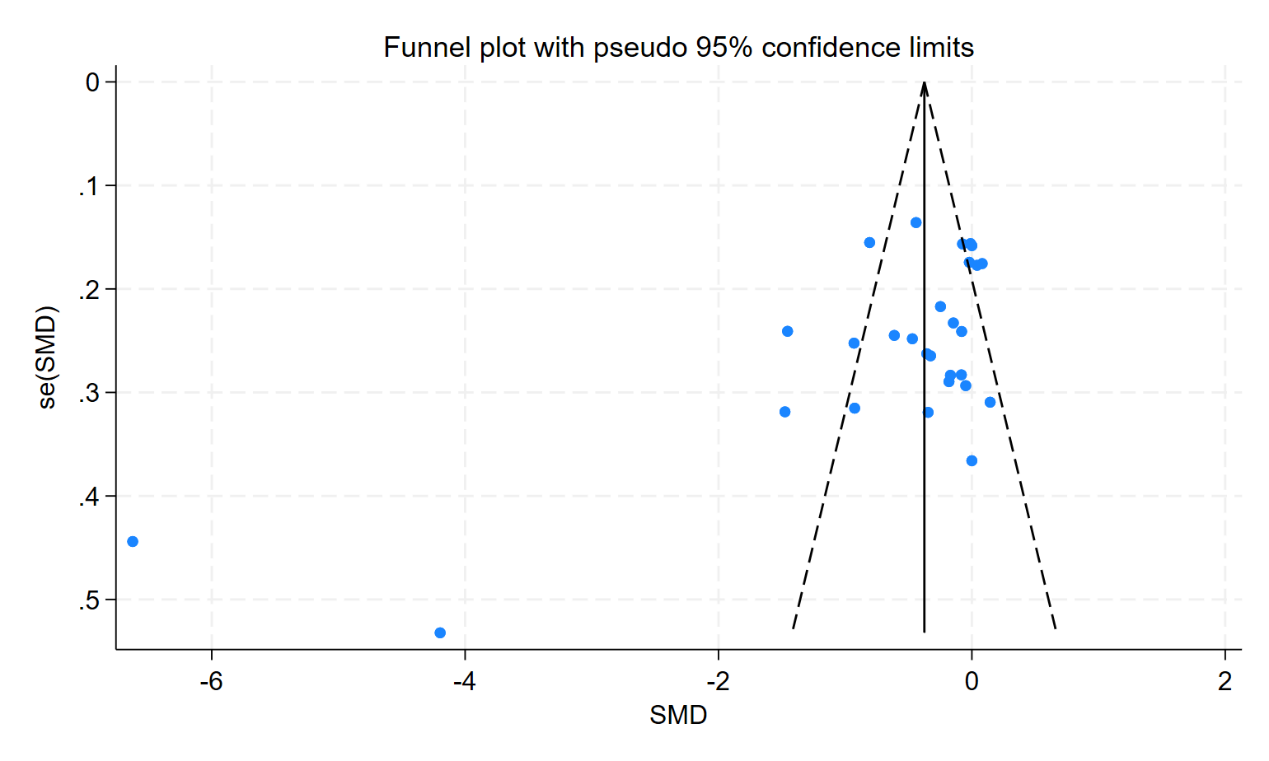


**Figure S3.** Funnel plot (anxiety)


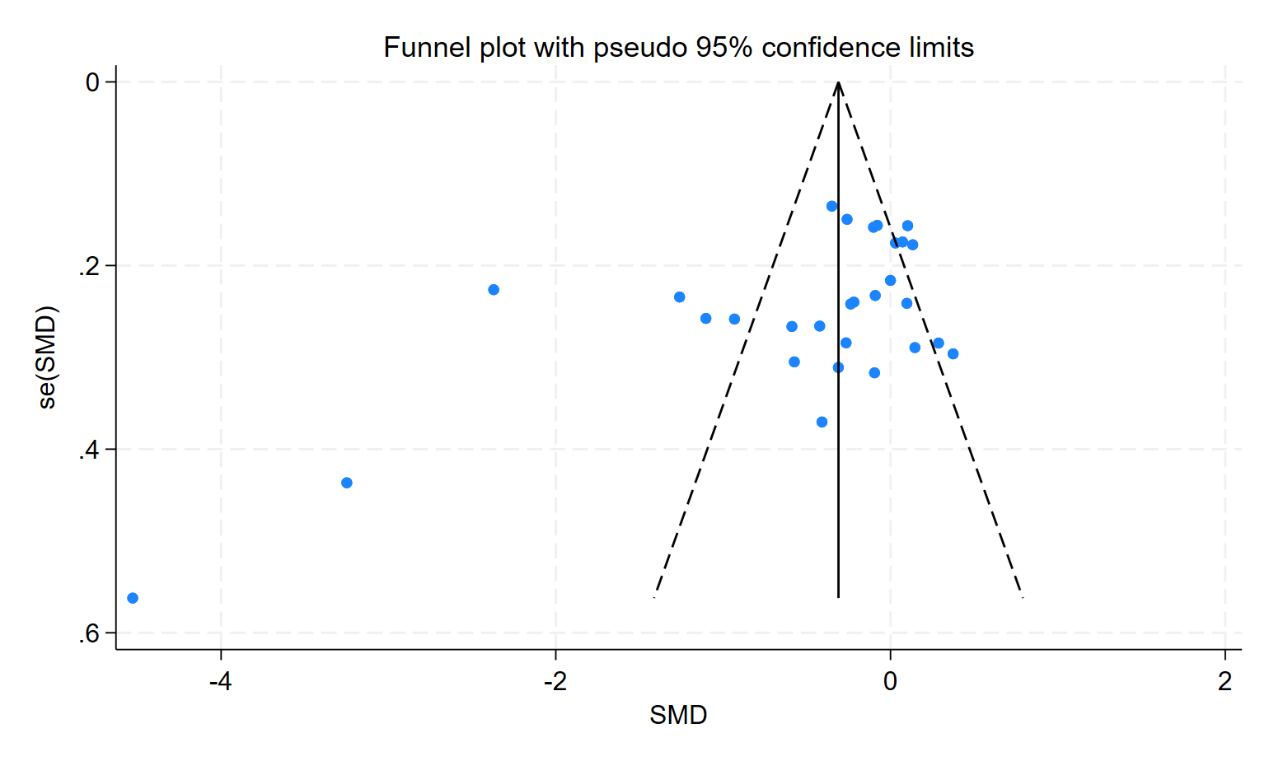


**Figure S4.** Sensitivity analysis results (depression)


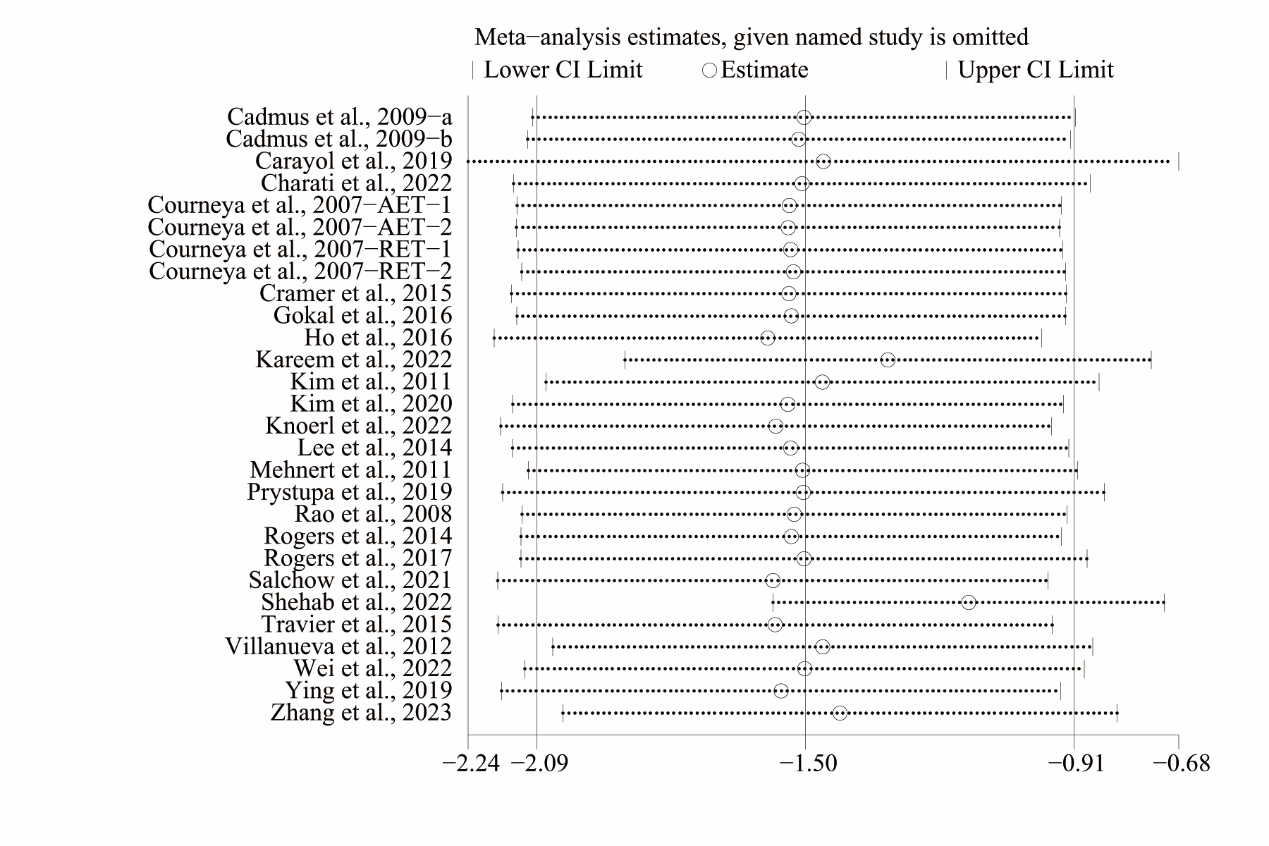


**Figure S5.** Sensitivity analysis results (anxiety)


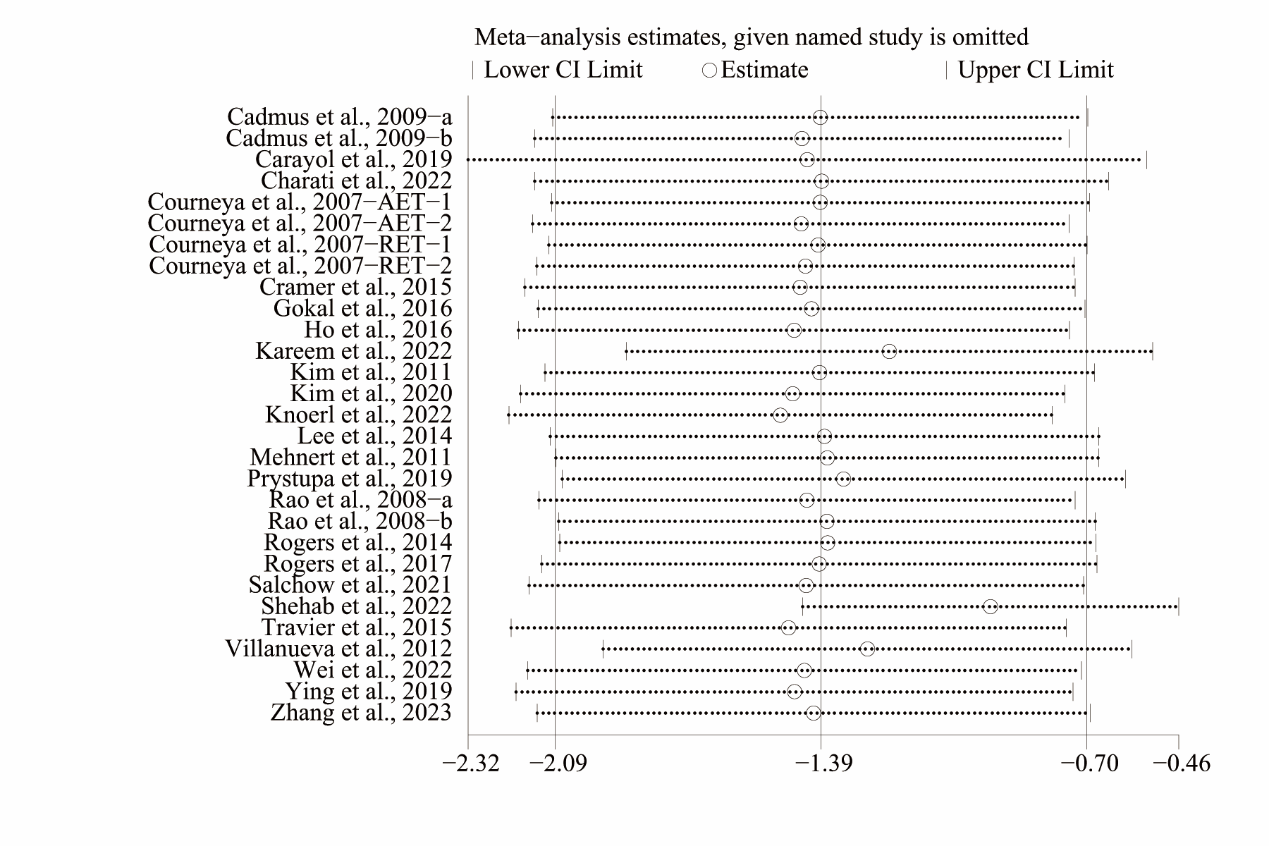


**Table S1.** Results of Egger’s test (depression)

| **Std_EFF** | **Coef.** | **Std. Err.** | **t** | ***p* > \|t\|** | **95% CI** |
| --- | --- | --- | --- | --- | --- |
| Slope | 0.800072 | 0.4288086 | 1.87 | 0.073 | -0.0813567, 1.681501 |
| Bias | -5.575751 | 1.936401 | -2.88 | 0.008 | -9.556081, -1.595421 |

**Abbreviations:** Coef, coefﬁcient; Std. Err, standard error; t, *t*-test statistic; *p*, probability; 95% CI, 95% Conﬁdence Interval.

**Table S2.** Results of Egger’s test (anxiety)

| **Std_EFF** | **Coef.** | **Std. Err.** | **t** | ***p* > \|t\|** | **95% CI** |
| --- | --- | --- | --- | --- | --- |
| Slope | 0.6279302 | 0.3791171 | 1.66 | 0.109 | -0.1499538, 1.405814 |
| Bias | -4.478931 | 1.725285 | -2.60 | 0.015 | -8.018923, -0.938938 |

**Abbreviations:** Coef, coefﬁcient; Std. Err, standard error; t, *t*-test statistic; *p*, probability; 95% CI, 95% Conﬁdence Interval.
